# Periodontal regenerative effect of enamel matrix derivative in diabetes

**DOI:** 10.1371/journal.pone.0207201

**Published:** 2018-11-15

**Authors:** Kohei Takeda, Koji Mizutani, Takanori Matsuura, Daisuke Kido, Risako Mikami, Masahiro Noda, Prima Buranasin, Yoshiyuki Sasaki, Yuichi Izumi

**Affiliations:** 1 Department of Periodontology, Graduate School of Medical and Dental Sciences, Tokyo Medical and Dental University (TMDU), Tokyo, Japan; 2 Medical Innovation Promotion Center, Institute of Research, Tokyo Medical and Dental University (TMDU), Tokyo, Japan; Università degli Studi della Campania, ITALY

## Abstract

The present study aimed to investigate the periodontal regenerative effect of enamel matrix derivative (EMD) in diabetes. Thirty-six rats were assigned to streptozotocin-induced diabetes or control (non-diabetic) groups. Three-wall intrabony defects were surgically generated in the bilateral maxilla molar, followed by application of EMD or saline. Primary wound closure and defect fill were evaluated via histomorphological analysis and micro-computed tomography. mRNA expression levels of inflammatory and angiogenic factors in the defects were quantified via real-time polymerase chain reaction. Gingival fibroblasts were isolated from control animals and cultured in high-glucose (HG) or control medium. The effects of EMD on insulin resistance and PI3K/Akt/VEGF signaling were evaluated. The achievement rate of primary closure and the parameters of defect fill were significantly higher at EMD-treated site than at EMD-untreated sites in both diabetic and non-diabetic rats, although defect fill in the diabetic groups was significantly lower in the control groups on two-way repeated-measures analysis of variance (for both, *p*<0.05). Newly formed bone and cementum were significantly increased at EMD-treated sites in diabetic rats than at EMD-untreated sites in control rats (for both, *p*<0.05). *Vegf* was significantly upregulated at EMD-treated sites in both diabetic and non-diabetic rats (for both, *p*<0.05). *In vitro*, insulin or EMD-induced Akt phosphorylation was significantly lower in cells cultured in HG medium (*p*<0.05). EMD-mediated *Vegf* upregulation was suppressed by the Akt inhibitor wortmannin, although the effect was significantly lower in HG medium (*p*<0.01). In conclusion, EMD might promote periodontal tissue regeneration via Akt/VEGF signaling, even in a diabetic condition.

## Introduction

Diabetes mellitus (DM) is a metabolic disease characterized by chronic hyperglycemia. Patients with diabetes have a high prevalence and rate of progression of periodontal disease because of their increased susceptibility to infection [1], and evidence for a bidirectional link between DM and periodontal disease has accumulated in recent years [2–5]. DM exacerbates periodontal disease by enhancing osteoclastogenesis and increasing apoptosis among osteoblasts [6]. Healing after dental treatment is impaired in patients with diabetes, since wound healing is defective in the diabetic condition owing to impairment of neutrophil activation and responses, fibroblast migration and proliferation, and angiogenesis [7, 8]. In addition, poor responses to periodontal treatment have been reported in such patients [9, 10]. We previously hypothesized that insulin resistance in the periodontal tissue is involved in delayed and impaired wound healing and subsequently confirmed that insulin resistance was induced in periodontal tissues, similar to induction in the livers and aortas of obese rodents [11]. Furthermore, a recent study reported delayed wound healing in streptozotocin (STZ)-induced diabetic rats caused by fibroblasts migration and proliferation disorders due to impaired insulin signaling via the phosphoinositide 3-kinase (Pl3K)/Akt pathway [12].

An enamel matrix derivative (EMD) has been extracted from developing porcine teeth. EMD comprises over 90% of protein complex which is comprised of an amelogenin. On investigating the mechanisms whereby EMD induces periodontal regeneration, numerous *in vitro* studies used various cellular systems, showing that EMD promotes the differentiation of osteogenic precursors [13], enhances proliferation and matrix production among periodontal ligament cells [14, 15], and inhibits epithelial cell proliferation [16]. Furthermore, EMD stimulates angiogenesis by activating endothelial cells and regulates osteogenesis [17]. Accordingly, EMD has been most widely used for periodontal regenerative therapy [18] and effectively promotes regeneration of the alveolar bone, cementum, and periodontal ligaments in the bone defect with periodontally-involved teeth [15, 19]. However, few studies have investigated periodontal tissue regeneration by EMD in the diabetic condition. Moreover, clinical studies examining periodontal tissue regeneration in patients with diabetes are lacking, given the ethical considerations of conducting prospective clinical trials [20]. Streptozocin (STZ) is a glucosamine-nitrosourea used clinically as a chemotherapeutic agent to treat pancreatic β cell carcinoma. STZ effectively destroys pancreatic β cells and causes hyperglycemia [21]. Rodent models of STZ-induced hyperglycemia are often used for studies on diabetes and diabetic complications. Therefore, in this study, we investigated whether insulin-deficient diabetes may inhibit the wound healing process related to the hyperglycemia-induced insulin resistance. A previous study reported that the EMD application sites in STZ-induced diabetic model rats had significantly shorter junctional epithelium lengths than non-application sites [22]. However, the ability of EMD to affect periodontal wound healing and tissue regeneration including bone, cementum, and periodontal ligament formation in DM is still unclear, as is the underlying mechanism, owing to the lack of studies on the effects of EMD *in vitro* and *in vivo*.

Therefore, this study aimed to investigate the regenerative effect of EMD on intrabony defects in rat models of diabetes. We explored the potential mechanism underlying this effect by examining the expression of insulin signaling-related angiogenesis markers via regulation of the PI3K/Akt pathway during the wound healing process *in vivo* and *in vitro*, using gingival fibroblasts cultured in high-glucose (HG) medium.

## Materials and methods

### Animals and induction of diabetes

**Fig 1:**
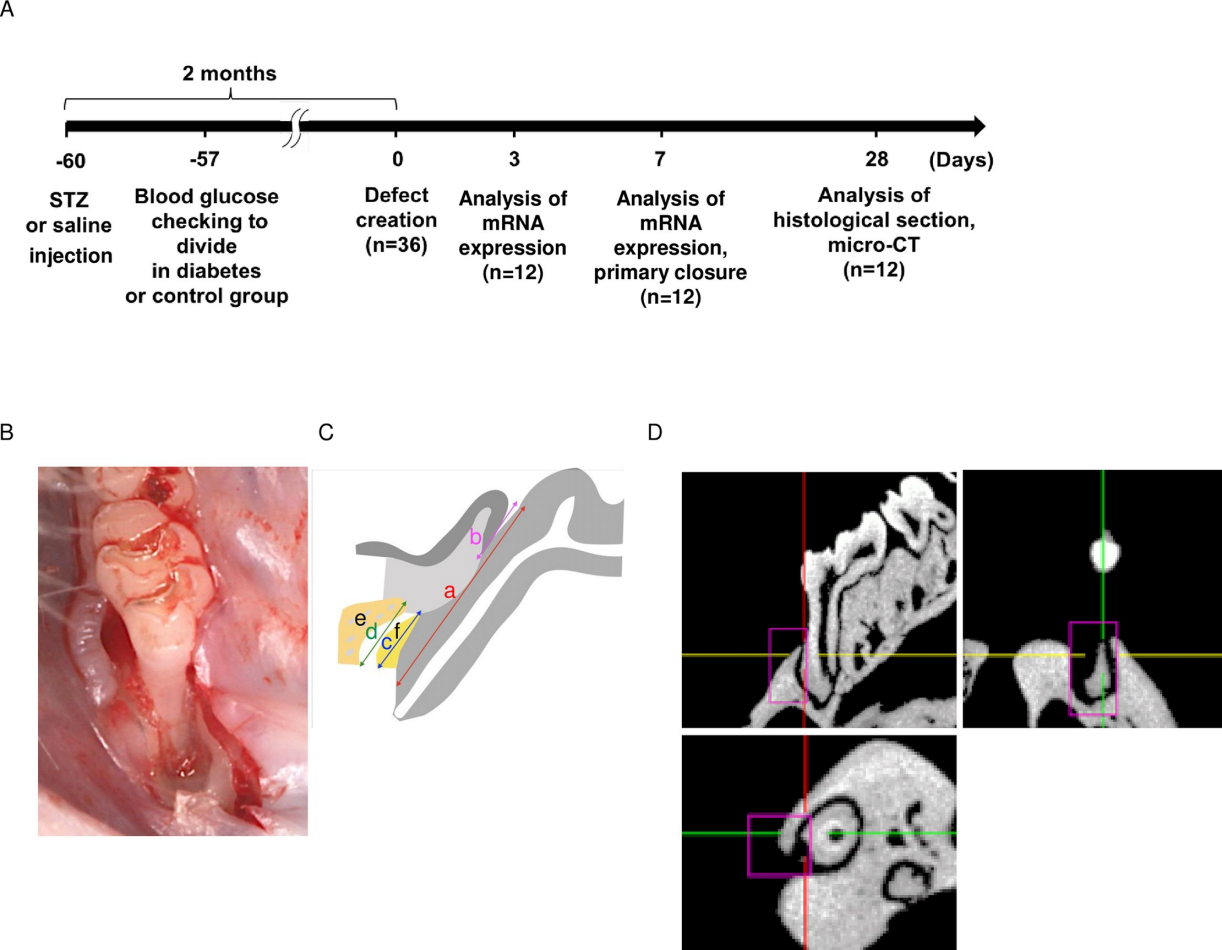
Study design. (A) Diabetes was induced via intraperitoneal injection of streptozotocin (STZ). Seventy-two hours after injection, rats with fasting glucose levels above 350 mg/dL were used as diabetic rats. In total, 36 animals (4 per group per time point) and 72 surgically induced defects (2 per animal) were evaluated. (B) Imaging of surgical defects. Intrabony defects were created bilaterally on the mesial surface of the maxillary first molar along the line of the maxillary dentition. (C) Histomorphometric measurement method at surgical sites. The distances from the lowest point of the defect to the most coronal point of the cement-enamel junction (CEJ) (a), junctional epithelium (b), newly formed cementum (c), and newly formed bone (d) were measured. The area of new bone (e) and cementum (f) were also measured. (D) The sections of a micro-computed tomographic image. Newly formed bone was three-dimensionally assessed by focusing on a boxed region of interest (pink-framed rectangles). The volume of bone defects was evaluated in a 1.0 × 1.0 × 2.0-mm box shape.

Six-week-old male Slc:Wistar rats (Sankyo Labo Service Corporation, INC., Tokyo, Japan) were used (n = 36) in this study. All animal experiments were approved by the Institutional Animal Care, Animal Research and Use Committee of Tokyo Medical and Dental University (No. A2018-217A). Fig 1A shows a schematic representation of the study design. Diabetes (n = 18) was induced via intraperitoneal injection of STZ (60 mg/kg in saline) after 12 h of fasting [23, 24]; rats in the control group (n = 18) were treated with the same volume of saline only. Body weights and plasma glucose levels were measured immediately before injection, 72 h after injection, and immediately before the surgical procedure. After 72 h, rats with fasting glucose levels above 350 mg/dL were considered diabetic rats [25]. Plasma insulin levels were determined using an enzyme-linked immunosorbent assay kit in accordance with the manufacturer’s instructions (Mercodia Rat Insulin ELISA, Mercodia, Oppland, Sweden).

### Surgical procedure

Intrabony defects were surgically induced 2 months after injection of STZ or saline. Animals were anesthetized with an intramuscular injection of 90 mg·L^-1^·kg^-1^ ketamine hydrochloride and 10 mg·L^-1^·kg^-1^ 2% xylazine. A crestal incision was made toward the mesial alveolar ridge to the first molar on both sides of the maxilla. Subsequently, the buccal and palatal flaps were raised to expose the mesial root of the first molar. Intrabony defects (1.0 × 1.0 × 2.0 mm) were then induced along the line of the maxillary dentition using a 0.5-mm-diameter bur. The exposed surface of the mesial root was debrided with an excavator to remove the cementum (Fig 1B). The root surfaces were rinsed with saline. EMD (Emdogain gel, Straumann, Switzerland) was then applied to the exposed root surface at the test sites, and control sites were left untreated. Finally, the flaps were closed with absorbable sutures (Vicryl 5–0; Ethicon Products, Tokyo, Japan).

### Observation of postsurgical healing

To evaluate wound closure, standardized intraoral digital photographs were captured immediately after the surgical procedure and at 7 days post-surgery at a fixed magnification (1.5×) from approximately 70° to the upper occlusal plane, with the mouth opened using Hashimoto’s mouth gag. The wound closure rate was evaluated on the basis of whether the root surfaces of the surgical sites were completely covered with the epithelium or partially exposed due to incomplete epithelization. Evaluations were independently made by two blinded examiners (T.M. and D.K.) for data analysis.

### Histological and histomorphometric analysis

The maxillae were harvested 28 days after surgery, fixed in 4% buffered formalin, and decalcified with 10% ethylenediaminetetraacetic acid. The tissue blocks embedded in paraffin were prepared for serial mesio-distal sections (thickness of 5 μm) with a microtome and stained with hematoxylin and eosin. Five central sections were selected per specimen for histomorphometric analysis using a light microscope. The following measurements were obtained for histomorphometric analysis (Fig 1C): cement-enamel junction to the bottom of the bone defect, length of the junctional epithelium, newly formed cementum, new bone, area of new bone, and area of new cementum. Linear measurements were obtained by digitally tracing the margins of areas using ImageJ software (National Institutes of Health, Bethesda, MD, USA). Measurements were independently obtained by two blinded examiners (T.M. and D.K.).

### Micro-computed tomographic (CT) scan analysis

Micro-CT analysis was performed to observe the newly formed mineralized tissue with the inspeXio SMX-100CT system (Shimadzu Science, Tokyo, Japan) at 75 kV and 140 μA. Using three-dimensional analysis software (TRI/3-D-BON, Ratoc System Engineering, Tokyo, Japan), micro-CT images were reconstructed to form three-dimensional images. Within the intrabony defects, the volume of interest was determined to be a cubic column (1.0 × 1.0 × 2.0 mm) excluding the original alveolar bone (Fig 1D). The new bone in the three-dimensional images and the following parameters of the columns were measured: (1) bone volume (BV), (2) cancellous bone volume (CBV), (3) bone mineral density (BMD), and (4) cancellous bone mineral content (CBM). Measurements were independently obtained by two blinded examiners (T.M. and D.K.).

### *In vivo* mRNA expression analysis

Soft tissue within the defects was harvested on 3 and 7 days after surgery. Samples were homogenized using Lysing Matrix A (MP Biomedicals, Santa Ana, CA, USA). After total RNA extraction from samples with RNeasy Fibrous Tissue Kit (Qiagen, Hilden, Germany) and reverse transcription, real-time quantitative polymerase chain reaction (PCR) was performed with SYBR Premix Ex Taq II (Takara, Shiga, Japan) to detect vascular endothelial growth factor (*Vegf*), collagen type I (*Col1*), *Runx2*, tumor necrosis factor-α (*Tnf-α*), and interleukin type 6 (*Il-6*) expression levels. Target mRNA expression levels were normalized to glyceraldehyde 3-phosphate dehydrogenase (*Gapdh*) expression levels. Primer sequences are listed in S1 Table.

### *In vitro* analysis of the insulin signaling pathway

For *in vitro* analysis, gingival fibroblasts were isolated from the maxillae gingiva of control rats, as described previously [12]. In brief, the gingiva, including the epithelium and connective tissue, was harvested from the control rats. After dispase I treatment, the connective tissue was minced into small pieces (2 mm^2^) and incubated in Dulbecco’s modified Eagle’s medium (D-MEM) with 10% fetal bovine serum (FBS) and 3% antibiotics at 37°C. Migrated fibroblast cells were used at passage 3–6 for experiments. The cells were cultured in a medium containing 15 mM glucose as the control or 50 mM glucose as the HG condition on the basis of our previous studies using gingival fibroblast in high-glucose medium [12, 26]. For osmotic control, mannitol was added as control for hyperosmolarity in the high-glucose groups.

To analyze the insulin signaling-related cell migration and proliferation via the PI3K and mitogen-activated protein kinase pathways, phosphorylated Akt (p-Akt) and Erk1/2 (p-Erk1/2) levels were evaluated following insulin stimulation (100 nM) via western blotting. Primary fibroblasts were cultured with the respective glucose concentrations in D-MEM containing 0.5% FBS at 37°C and then treated with insulin (100 nM) for an additional 10 min. Following insulin stimulation, the medium was aspirated and cells were washed with ice-cold PBS. Lysis buffer containing RIPA Buffer (Wako, Tokyo, Japan), protease inhibitor cocktail, and phosphatase inhibitor (Sigma) was added to the culture dish, and the cells were harvested via scraping with a cold scraper. The pellets were sonicated and centrifuged at 13,400 rpm for 10 min at 4°C. The supernatants, 4X Laemmli sample buffer (Bio-rad, Hercules, CA, USA), and 2-mercaptoethanol (Sigma) were mixed and boiled for 5 min at 95°C. Equal amounts of proteins were electrophoresed on Mini-Protean TGX gels (Bio-rad), at a constant voltage of 100 V, and electro-transferred to nitrocellulose membranes (Amersham) at a constant current of 200 mA. Membranes were incubated for blocking at room temperature (25°C) with Blocking Buffer (StartingBlock T20, Thermo Fisher Scientific, Waltham, MA, USA) for 30 min. Thereafter, the membranes were incubated overnight with a 1:1000 dilution of total- (Cell Signaling Technology, Danvers, MA, USA, #9272) and phospho-Akt (Cell Signaling Technology #4060) antibodies, and total- (Cell Signaling Technology #9102) and phospho-Erk1/2 antibodies (Cell Signaling Technology #4370) in Blocking Buffer at 4°C. The membranes were washed with PBST (80 mM Na_2_HPO_4_, 50 mM NaH_2_PO_4_, 100 mM NaCl, and 1% Tween 20) for 5 min and incubated with a 1 : 5,000 dilution of donkey antirabbit IgG-HRP (Santa Cruz Biotechnology, sc-2313, Dallas, TX, USA) secondary antibody in Blocking Buffer at room temperature for 1 h. Thereafter, membranes were washed intensively with PBST. The enhanced chemiluminescence reaction generated using the ECL Western Blotting Substrate (Pierce) was analyzed using Ez-Capture MG and analyzed using an imaging software (National Institutes of Health, Bethesda, MD, USA).

### *In vitro* EMD treatment

To investigate whether the effects of EMD occur via regulation of the PI3K/Akt/VEGF pathway, the p-Akt level was evaluated via western blotting. EMD powder (30 mg/vial) was dissolved in 1 mL of acetate buffer (pH 5) and diluted to 100 μg/mL in D-MEM containing 10% FBS [27]. Cells were then incubated in D-MEM with 100 μg/mL EMD to analyze Akt phosphorylation and *Vegf* expression. In addition, the cells were treated with the Akt inhibitor wortmannin (Wako Pure Chemical Industries, Tokyo, Japan) at 100 nM 30 min before EMD treatment [28].

### Statistical analysis

Data are expressed as mean ± standard deviation values. The chi-squared test was used to compare the frequency of primary closure in control and diabetic rats. In cases of one independent variable, such as analysis of the insulin signaling pathway, multiple-group comparisons were analyzed using one-way analysis of variance followed by the Tukey-Kramer post-hoc test. In cases of two independent variables, such as histomorphometric measurements, micro-CT analyses, and mRNA expression parameters *in vivo*, comparisons among multiple groups were analyzed using two-way repeated-measures analysis of variance. Diabetic status (diabetic or not) was a between-subjects factor, and application of EMD or saline was a within-subjects factor. P-values less than 0.05 were considered to represent statistically significant differences.

Regarding data reproducibility, each examiner (T.M. and D.K.) repeatedly measured each parameter via histomorphometric analysis and micro-CT analysis 24 h after the first measurements. The comparison between the examiners (inter-examiner reproducibility) and the comparison within the examiner (intra-examiner reproducibility) were determined using Pearson correlation analysis.

All statistical analyses were carried out in StatView (ver. 5.0, SAS Co. Ltd., Cary, NC).

## Results

### Systemic characteristics of control and DM rats

All animals survived during 2 months of diabetes induction and during the postoperative observation period. Rats in the DM group showed significantly decreased body weight (*p*<0.01) and plasma insulin levels (*p*<0.05) and significantly higher fasting blood glucose levels than those in the control group (*p*<0.01) (S2 Table).

### Observation of postsurgical healing

**Fig 2:**
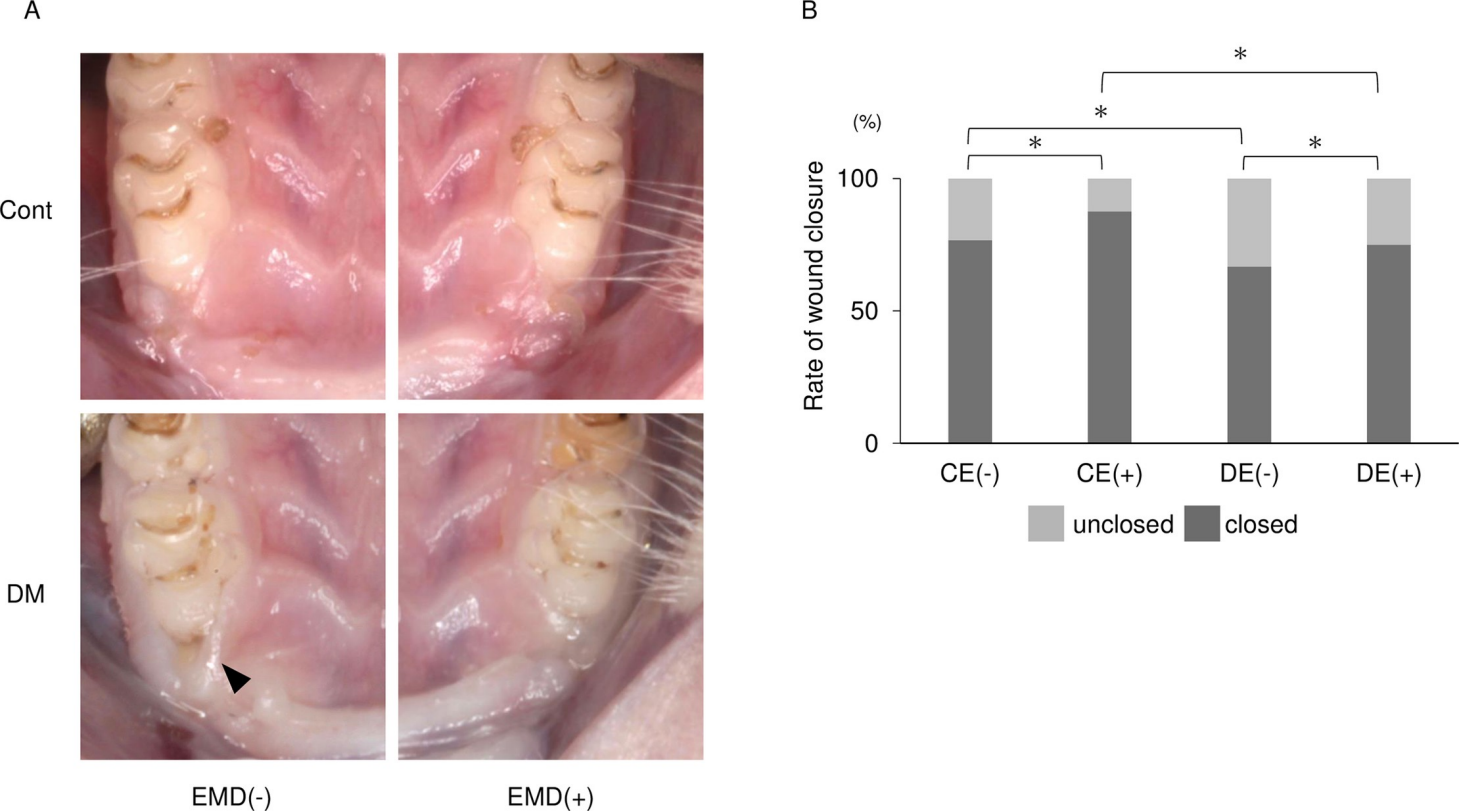
Analysis of postsurgical wound healing. (A) Representative photographs of postsurgical wounds at 7 d after surgery. In the EMD-untreated sites of diabetic rats, wound closure deficiency was observed (arrowheads). (B) Wound closure rates 7 d after surgery differed significantly among the four groups [control without EMD, CE(-); control with EMD, CE(+); diabetes without EMD, DE(-); and diabetes with EMD, DE(+)].*p < 0.05 (chi-squared test, n = 24).

At 7 days after surgery, wound closure rates were significantly higher in control rats than in DM rats (*p*<0.01). Wound closure rates were significantly higher at the EMD-treated sites of both control and diabetic rats than at untreated sites (*p*<0.01) (Fig 2).

### Histological and histomorphometric analysis

**Fig 3:**
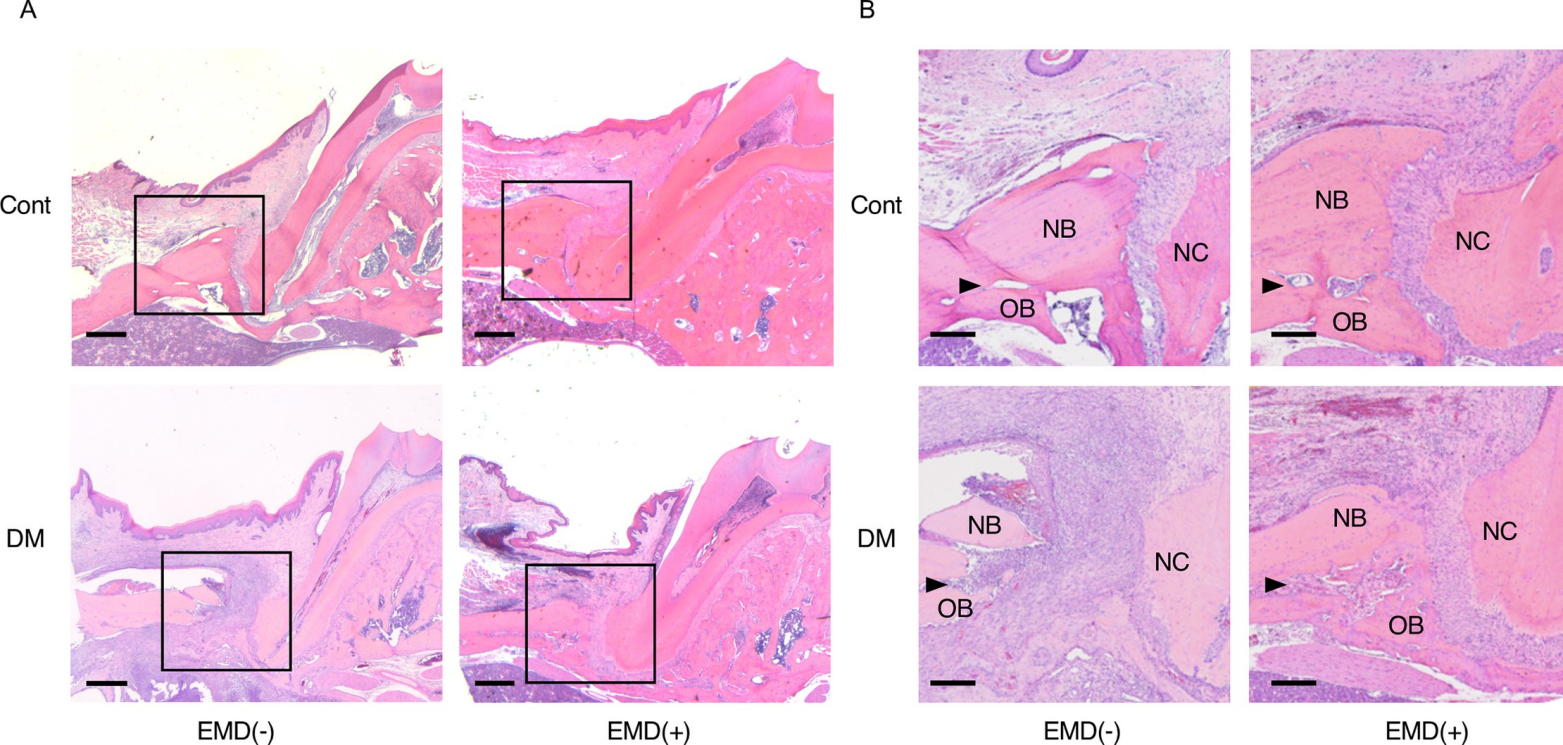
Histological analysis. (A) Representative histological photographs at 28 days after surgery of the four groups [control without the enamel matrix derivative [EMD] and without the cement enamel [CE(-)]; control with EMD, CE(+); diabetes without EMD, DE(-); and diabetes with EMD, DE(+)]. In the mesio-distal section, the defects were filled with newly formed connective tissue, cementum, and bone. Newly formed bone was observed along the roots of surgical defects. Hematoxylin–eosin staining, magnification 10×. Scale bar: 500 μm. (B) Higher magnification of the interface of the original bone (OB), newly formed bone (NB), and newly formed cementum (NC) at the bottom level of the original defect (arrowheads). Hematoxylin–eosin staining, magnification 40×. Scale bar: 100 μm.

The defects were filled with newly formed connective tissue, cementum, and bone, including abundant cementoblasts and osteoblasts along the root surface, after 28 days. Dense and vertical collagen fibers between the root surfaces and newly formed bone were observed in the control group both without and with EMD treatment [CE(-) and CE(+), respectively]. By contrast, sparse and oblique collagen fibers were observed in the DM groups both without and with EMD [DE(-) and DE(+), respectively]. In the CE(+) and DE(+) groups, the narrow spaces of the periodontal ligament were observed in association with substantial bone formation (Fig 3A and 3B). A thick cellular cementum in the newly formed bone was observed in the CE(+) and DE(+) groups (S1A Fig). In the CE(+) and DE(+) groups, many cells forming cell substrate on the bone defect sides aggregated to form a new bone (S1B Fig). Newly formed cementum and bone layers were detected in all groups because the boundaries between the original and newly formed cementum and bone could be distinguished (Fig 3A and 3B).

**Fig 4:**
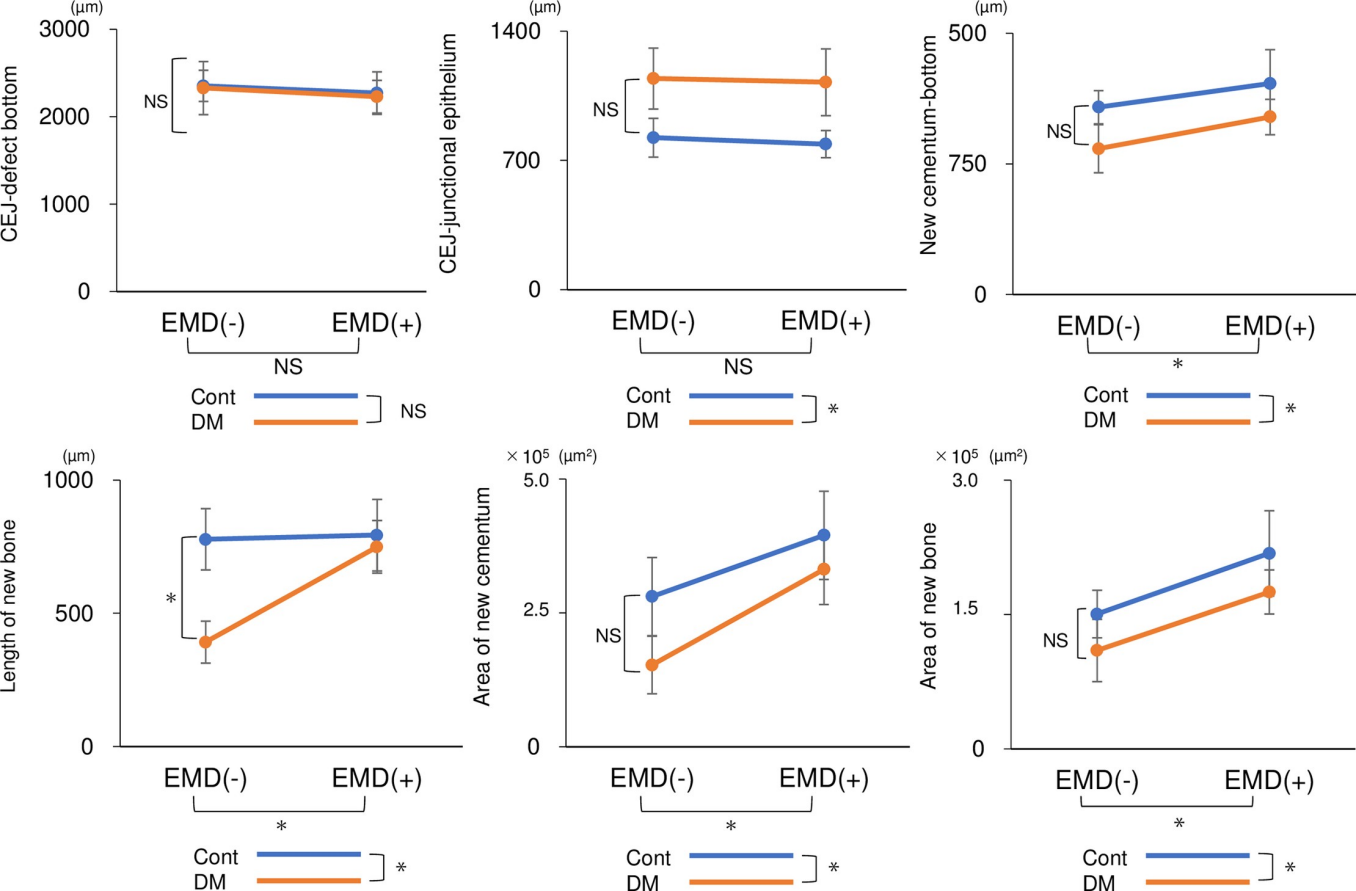
Histomorphometric analysis. The distance of the cement-enamel junction (CEJ) to the bottom of the defect, length of the junctional epithelium, length of new cementum, length of new bone, area of new bone, and area of new cementum were histomorphometrically compared among groups [control without EMD, CE(-); control with EMD, CE(+); diabetes without EMD, DE(-); and diabetes with EMD, DE(+)]. Measurements were independently obtained by two blinded examiners (T.M. and D.K.), and the data were analyzed. *p < 0.05 (two-way repeated-measures analysis of variance, n = 24). NS, not statistically significant.

The results of histomorphometric analysis are shown in Fig 4 and S3 Table. There was no significant difference in the distance from the cement-enamel junction to the bottom of the original defect in any group, indicating that the defects were induced uniformly. Newly formed connective tissue attachment and bone were increased at EMD-treated sites. Significant differences in the length of the junctional epithelium were detected in the between-subjects analysis, whereas application of EMD produced no significant difference in the within-subjects analysis. However, significant differences in the length of the newly formed cementum and in the area of the newly formed cementum and bone were detected upon within-subjects analysis and in the between-subjects analysis. Significant differences in the length of new bones were also detected between the CE(-) and CE(+) groups and between the CE(+) and DE(+) groups, showing that both the EMD treatment and diabetic status in rats with EMD-treated defects significantly influenced bone regeneration. Therefore, two-way repeated-measures analysis of variance revealed a simple main effect with significant differences detected between CE(-) and DE(-) and between DE(-) and DE(+).

The high stability of each parameter was confirmed by calculating the Pearson correlation coefficients (intra-examiner reproducibility: *p* < 0.05, r = 0.97 for T.M. and *p* < 0.05, r = 0.93 for D.K.; inter-examiner reproducibility: *p* < 0.01, r = 0.99).

### Micro-CT scan analysis

**Fig 5:**
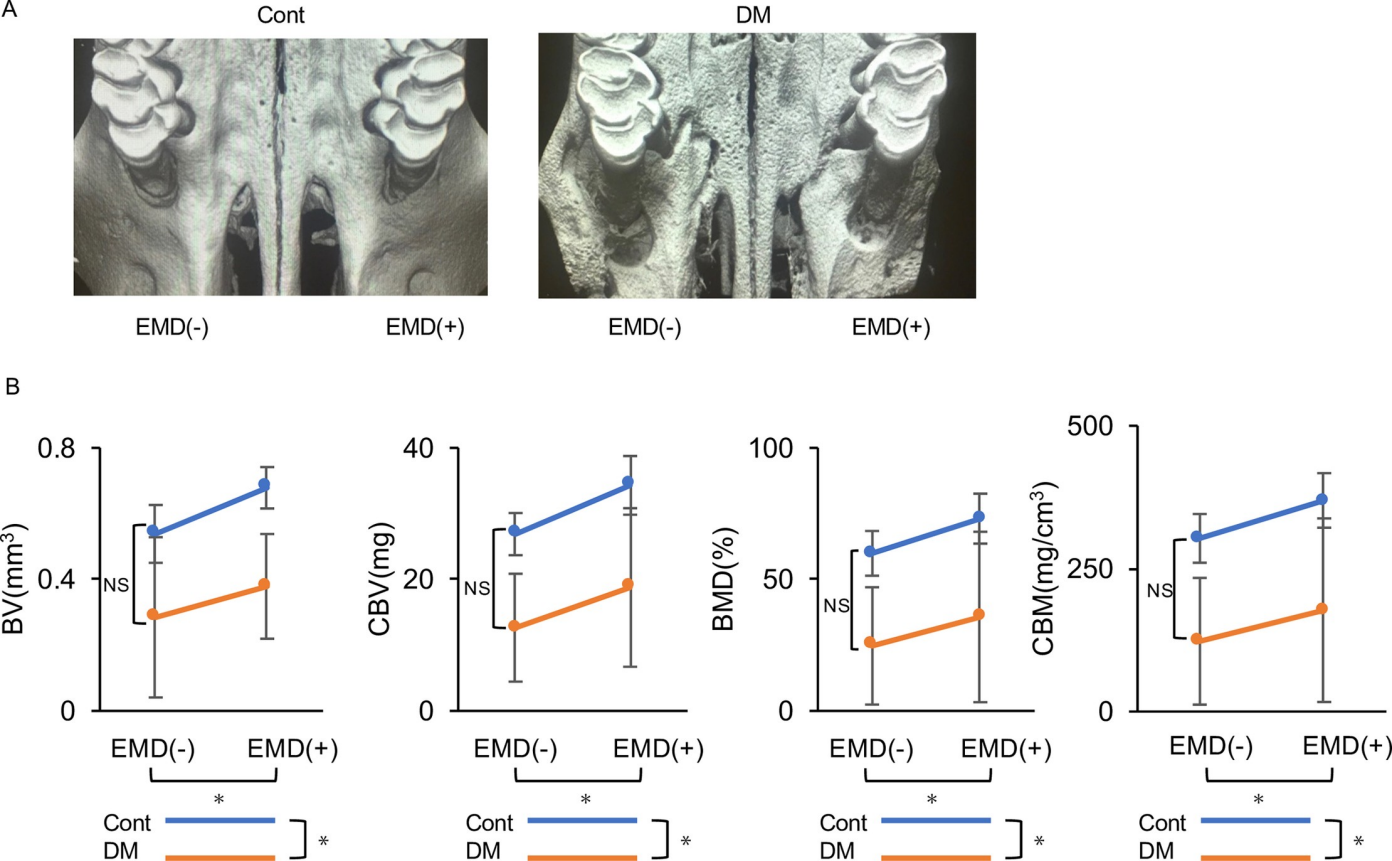
Micro-computed tomographic (CT) analysis. (A) Representative three-dimensional volume images of new bone formation by micro-CT after 28 d in the four groups [control without the enamel matrix derivative [EMD] and without the cement enamel CE(-); control with EMD, CE(+); diabetes without EMD, DE(-); and diabetes with EMD, DE(+)]. Defects in the control and DM groups were assessed by micro-CT. Less regeneration was observed in the DE(-) and DE(+) groups than in the CE(-) and CE(+) groups. More newly formed bone was observed in CE(+) and DE(+) than in CE(-) and DE(-). (B) Quantitative analysis of new bone formation by micro-CT in each group. (1) Bone volume, BV; (2) cancellous bone volume, CBV; (3) bone mineral density, BMD; and (4) cancellous bone mineral content, CBM were compared between groups. Measurements were independently made by two blinded examiners (T.M. and D.K.), and the data were analyzed. Data are presented as means ± SD (n = 24). *p < 0.05 (two-way repeated-measures analysis of variance). NS, not statistically significant.

Micro-CT analysis 28 days after surgery revealed that the extent of the defect fill was lower in the DE(-) and DE(+) groups compared with that in the CE(-) and CE(+) groups (Fig 5A). BV, CBV, BMD, and CBM were significantly lower in the DE(-) and DE(+) groups than in the CE(-) and CE(+) groups (for all, *p*<0.05) (Fig 5B and S4 Table). However, application of EMD had a significant positive effect on BV, CBV, BMD, and CBM (for all, *p*<0.05) (Fig 5B and S4 Table).

The high stability of each parameter was confirmed by calculating Pearson correlation coefficients (intra-examiner reproducibility: *p* < 0.05, r = 0.94 for T.M. and *p* < 0.05, r = 0.99 for D.K.; inter-examiner reproducibility: *p* < 0.01, r = 0.99).

### *In vivo* mRNA expression

**Fig 6:**
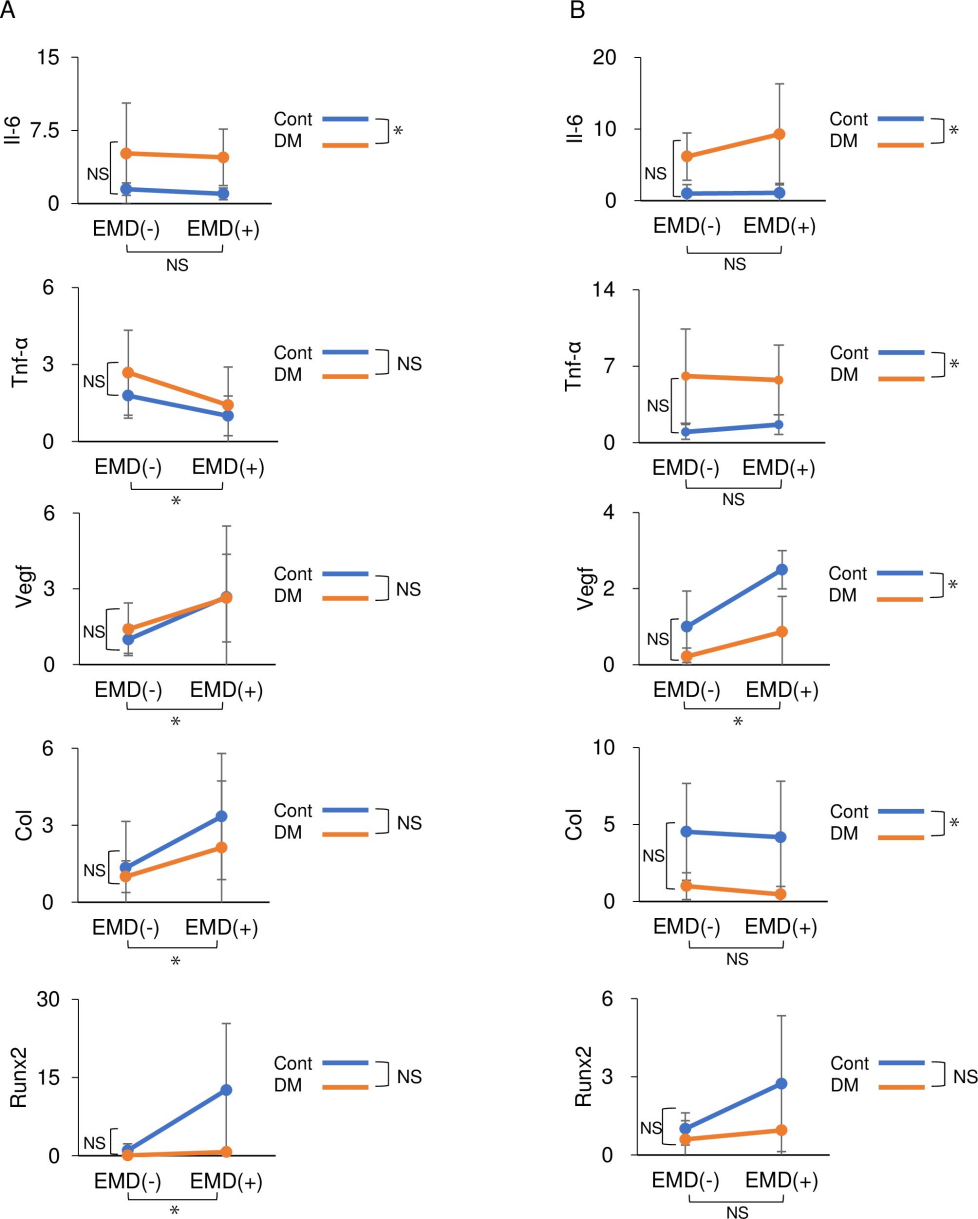
*In vivo* mRNA expression of inflammatory and angiogenic factors. (A) The mRNA expression levels of *Il-6*, *Tnf-α*, *Vegf*, *Col1*, and *Runx2* were determined via real-time quantitative polymerase chain reaction at 3 d and (B) 7 d. Inflammatory cytokines; *Il-6 and Tnf-α* were significantly upregulated in the DE(-) and DE(+) groups compared to those in the CE(-) and CE(+) groups after 7 d (*p*<0.05). Angiogenesis-related gene; *Vegf* were significantly upregulated in the CE(+) and DE(+) groups after 3 and 7 d (3 d *p*<0.01, 7d *p*<0.05). *Col1* was significantly upregulated in the CE(+) and DE(+) groups compared to those in the CE(-) and DE(-) groups after 3 d (*p*<0.05). These findings were confirmed in three independent experiments. Data are presented as mean ± SD values (n = 12). *p < 0.05 (two-way repeated-measures analysis of variance). NS, not statistically significant.

*Il-6* was significantly upregulated in the DE(-) and DE(+) groups compared to that in the CE(-) and CE(+) groups 3 and 7 days after surgery (3 day *p*<0.05, 7day *p*<0.01) (Fig 6A and 6B). *Tnf-α* was significantly upregulated in the DE(-) and DE(+) groups compared to that in the CE(-) and CE(+) groups 7 day after surgery (*p*<0.05) (Fig 6B). *Vegf*, which is associated with angiogenesis, was significantly upregulated in the CE(+) and DE(+) groups compared to that in the CE(-) and DE(-) groups on 3 and 7 days after surgery (3 day *p*<0.01, 7day *p*<0.05) (Fig 6A and 6B). In contrast, *Col1* was significantly upregulated in the CE(+) and DE(+) groups compared to that in the CE(-) and DE(-) groups after 3 day (*p*<0.05) (Fig 6A) and significantly upregulated in the CE(-) and CE(+) groups compared to that in the than in the DE(-) and DE(+) groups after 7 day (*p*<0.01) (Fig 6B). Furthermore, *Runx2* was significantly upregulated in the CE(-) and CE(+) groups than in the DE(-) and DE(+) groups after 3 day (*p*<0.05) (Fig 6A).

### Effects of EMD treatment on Akt phosphorylation and VEGF expression

Insulin-stimulated p-Akt was significantly elevated in fibroblasts cultured in both normal and HG media (for both *p*<0.05), however, the effect was more pronounced in cells cultured in media containing normal glucose levels. In addition, p-Erk levels were suppressed in fibroblasts cultured in HG medium compared to those in fibroblasts maintained in normal glucose medium (S2 Fig).

**Fig 7:**
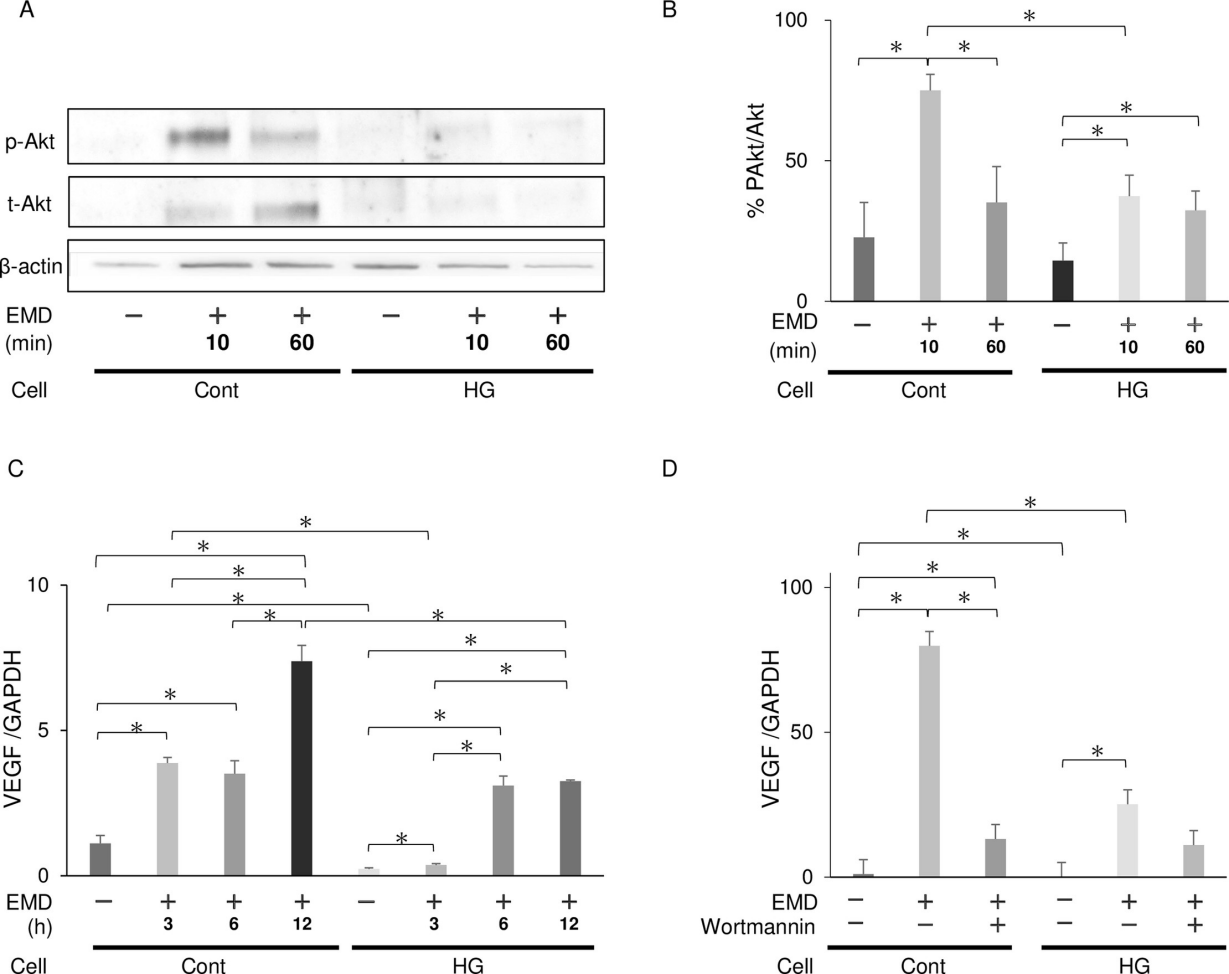
Akt and Erk1/2 phosphorylation in fibroblasts *in vitro*. (A) Representative immunoblot images of EMD-treated or -untreated groups. Effect of 10 or 60 min of EMD treatment on Akt phosphorylation in gingival fibroblasts maintained at a control (Cont) or high glucose (HG) concentration. (B) Akt phosphorylation was quantified via densitometric analysis and expressed as a percentage of phosphorylation in EMD-untreated groups. Data are presented as mean ± SD values. *p < 0.05 (Tukey-Kramer test). These findings were confirmed in three independent experiments. (C) The mRNA expression level of *Vegf* was measured via real-time polymerase chain reaction (PCR) in the four groups (control glucose medium without EMD, Cont-; control glucose medium with EMD for 3–12 h, Cont+; high glucose medium without EMD, HG-; and high glucose medium with EMD for 3–12 h, HG+). Data are presented as mean ± SD values. *p < 0.05 (Tukey-Kramer test). These findings were confirmed in three independent experiments. (D) The mRNA expression level of *Vegf* with/without wortmannin was measured via real-time PCR. Data are presented as mean ± SD values. *p < 0.05 (Tukey-Kramer test). These findings were confirmed in three independent experiments.

Akt phosphorylation was also significantly elevated in cells cultured in control and HG medium with EMD (Cont+ and HG+, respectively) (for both *p*<0.05). However, p-Akt levels in cells treated with EMD for 10 min were significantly upregulated in the Cont+ group compared with that in the HG+ group, with a 49.9% difference (*p*<0.01) (Fig 7A and 7B).

*Vegf* was significantly temporally upregulated after EMD treatment in both groups (for both *p*<0.05), with and without EMD, being significantly greater in the Cont+ group than in the HG+ group, even after 3 h of EMD treatment (*p*<0.05) (Fig 7C). *Vegf* was significantly downregulated by 37.42 ± 7.44% in both groups after wortmannin application (for both *p*<0.05) (Fig 7D).

## Discussion

To our knowledge, this is the first study demonstrating that EMD promotes wound healing and tissue regeneration *in vitro* and *in vivo* even in the diabetic condition. Patients with diabetes are known to have impaired therapeutic outcomes after non-surgical periodontal treatment [9, 10, 29, 30] and after guided tissue regeneration [31]. To date, the mechanisms underlying the association between periodontitis and DM have been evaluated on the basis of the augmentation of periodontal tissue destruction in studies on damage induced by advanced glycation end products (AGEs) [32], alteration of immune function [33], and induction of vascular dysfunction [34]. *In vivo* studies report that AGEs are involved in the mechanism underlying the healing of impaired periodontal tissue and osteogenesis in diabetic rats [35]. In the present study, although the effect of EMD on periodontal tissue regeneration was reduced, it was detected even in animals with uncontrolled diabetes.

STZ-induced animals are widely used for studies on diabetic complications following a 1–3-month diabetic duration [36], since STZ effectively destroys pancreatic β cells and causes hyperglycemia [21]. Loss of glucose transporters on pancreatic β cell membranes in humans and rodents is associated with hyperglycemia and precedes the development of insulin resistance [37]. In the present study, STZ-induced rats showed significant loss of body weight, hyperglycemia, insulin deficiency, increased systemic oxidative stress, and elevated inflammatory markers such as *Tnf-α* and *Il-6* in the gingiva 2 months after STZ injection. Previous studies report that microvascular blood flow preceding pancreatitis is increased in type 1 diabetes [38], and the level of *Il-1β* in the pancreas is locally elevated [39]. The level of *Il-1β* and *Tnf-α* in the gingiva of type 1 diabetes patients with periodontitis is also elevated [40]. In the present study, inflammation was considered to be caused locally in the gingiva and *Tnf-α* and *Il-6* were upregulated. Systemic and local oxidative stress induced by hyperglycemia for a 2-month diabetic duration produced insulin resistance in gingival fibroblasts and delayed gingival wound healing [12]. For periodontal regeneration of a rodent model, a surgically induced intrabony defect is considered to be reproducible [22, 41, 42]. Therefore, we decided to surgically prepare the critical-size bony defects 2 months after STZ injection, even though previous studies have used short-term hyperglycemic rats [22, 42].

In this experiment, primary wound closure after 7 day was satisfactory. The CE(+) group showed the highest ratio of primary closure, suggesting that EMD promotes soft-tissue wound healing. This finding is consistent with previous studies reporting that EMD also enhanced the proliferation of gingival fibroblasts [13, 43]. Interestingly, EMD accelerated wound healing in diabetic animals: the DE(+) group showed a significantly higher ratio than the DE(-) group, and the score was comparable to that of the CE(-) group.

Histomorphological analysis revealed that the periodontal regenerative parameters in the DE(+) group were significantly decreased compared to those in the CE(+) group. The results of histological analysis were similar to the three-dimensional results of micro-CT analysis. BV was decreased by 45% in the diabetic animals compared to controls without EMD treatment, concurrent with a previous clinical report wherein bone healing was compromised in patients with diabetes rather than in healthy patients [44]. The reduced bone regeneration observed in diabetic animals, regardless of EMD treatment, is attributed to the fact that primary closure is a crucial factor for tissue regeneration. In addition to the reduced tissue regeneration observed in the diabetic animals, lower expression levels of angiogenic and osteogenic markers were observed in the early healing phases, with and without EMD treatment. Since these markers are upregulated via the PI3K/Akt pathway, this result is consistent with insulin resistance impairing Akt activation in the HG condition in the *in vitro* experiments.

Interestingly, the regenerative effects of EMD were also partly observed in diabetic rats. The length of the junctional epithelium in the DE(+) group was 30% that of its length in the DE(-) group, consistent with previous reports [22, 45, 46]. Moreover, the height of newly formed bone in the DE(+) group was the same as that in the CE(-) group. The positive effects of EMD were also observed three-dimensionally via micro-CT, demonstrating a 26% and 33% increase in BV in non-diabetic and diabetic rats, respectively. Similar results were observed for CBV. This periodontal tissue regeneration in the DM groups may have resulted from EMD-mediated upregulation of angiogenic factors. A previous study reported that EMD induces *VEGF* expression in human gingival fibroblasts [27] and periodontal ligament cells [47]. In the present study, EMD treatment also upregulated *Vegf in vivo* and *in vitro*.

High glucose concentrations affect cell function on the basis of cell, tissue, and organ type. Blood glucose levels in healthy and STZ-induced diabetic rats are approximately 7 mM and 26 mM, respectively. However, the cell culture conditions occasionally differ from *in vivo* physiological conditions; the optimal glucose concentration for cultured gingival fibroblasts proliferation and migration was reported to be 15 mM or 20 mM [12, 48]. Our recent *in vitro* study reported that 72 h of exposure to 50 mM glucose concentration induced oxidative stress and impaired migration and proliferation in human gingival fibroblasts [26]. Thus, to clarify the effect of high glucose concentrations on the cultured gingival fibroblasts, we selected 50 mM as the highest acceptable concentration. Analysis of the insulin signaling pathway revealed that Akt-specific insulin resistance was induced by the HG medium. Moreover, an effect of EMD was observed in fibroblasts cultured in HG medium. Furthermore, p-Akt levels were significantly elevated in EMD-treated fibroblasts and inhibited by wortmannin, suggesting that EMD affected fibroblasts even in the HG medium via the Akt/VEGF pathway.

Overall, *in vivo* analysis revealed that periodontal tissue and bone regeneration were decreased in DM rats. However, the regenerative effects of EMD were also detected in DM rats. Based on the parallel slopes of the graphs of two-way repeated-measures analysis of variance (Figs 4–6), it was concluded that EMD yielded comparable effects regardless of diabetic status *in vivo*. The present results suggest that the clinical application of EMD may have some beneficial effects, even in diabetes. The present results indicate that EMD application is highly beneficial for periodontal tissue regeneration via the Akt-VEGF pathway even in the diabetic condition.

## Supporting information

S1 Fig Histological observation. (A) Representative photographs of histological analysis of the four groups [control without enamel matrix derivative [EMD] and without the cement enamel [CE(-)]; control with EMD, CE(+); diabetes without EMD, DE(-); and diabetes with EMD, DE(+)]. Higher magnification images of the interface shown in Fig 3, focusing on the coronal ends of newly formed cementum. Hematoxylin–eosin staining, magnification 100×. Scale bar: 10 μm. (D) Higher magnification images of the interface shown in Fig 3, focusing on the coronal ends of newly formed bone. Hematoxylin–eosin staining, magnification 100×. Scale bar: 10 μm.
(PPTX)

S2 Fig Analysis of the insulin signaling pathway. (A) Effect of insulin on Akt and Erk1/2 phosphorylation in gingival fibroblasts cultured in Cont and HG medium. Representative immunoblots of lysates from insulin-treated and -untreated gingival fibroblasts are shown. (B) Akt and Erk1/2 phosphorylation was quantified via densitometric analysis and expressed as a percentage of phosphorylation in insulin-untreated gingival fibroblasts. Insulin-induced Akt phosphorylation was significantly decreased in the HG medium; however, Erk1/2 phosphorylation was increased. Data are presented as mean ± SD values. *p < 0.05 (Tukey-Kramer test). These findings were confirmed in three independent experiments.
(DOCX)

S3 Fig Original membrane in Fig 7. The membrane fragment including the 60 kD band was treated with total and phosphorylated Akt. B-a. Membrane in Fig 7 as p-Akt, B-b Membrane in Fig 7 as t-Akt. C. Membrane in Fig 7 as β-actin.
(PPTX)

S4 Fig Original membrane in S2 Fig. The membrane was separated at 50 kD. The membrane fragment including the 60 kD band was treated with total and phosphorylated Akt. The other fragment including at 42,44, and 45 kD fragments was treated with total and phosphorylated Erk1/2 and β-actin. A. Original membrane in S2 Fig, B-a. Membrane in S2 Fig as t-Erk1/2, B-b. Membrane in S2 Fig as p-Erk1/2, C-a. Membrane in S2 Fig as t-Akt, C-b Membrane in S2 Fig as p-Akt. D. Membrane in S2 Fig as β-actin.
(PPTX)

S1 Table Sequences of primers used for reverse-transcription real-time quantitative polymerase chain reaction. (DOCX)

S2 Table Changes in body weight, fasting blood glucose level, plasma insulin level, and urine 8-hydroxy-2-deoxyguanosine levels in rats after streptozotocin treatment (60 mg/kg). (DOCX)

S3 Table Statistical results of histological analysis. (DOCX)

S4 Table Statistical results of micro-computed tomographic analysis. (DOCX)
