# Correction: Periodontal regenerative effect of enamel matrix derivative in diabetes

**DOI:** 10.1371/journal.pone.0218798

**Published:** 2019-06-18

**Authors:** Kohei Takeda, Koji Mizutani, Takanori Matsuura, Daisuke Kido, Risako Mikami, Masahiro Noda, Prima Buranasin, Yoshiyuki Sasaki, Yuichi Izumi

In the Abstract, there is an error in the eighth sentence. The correct sentence is: The parameters of defect fill were significantly higher at EMD-treated site than at EMD-untreated sites in both diabetic and non-diabetic rats.

Fig 2 is incorrect. The right side of the figure should not contain any statistically significant difference symbols. There is an error in the caption for Fig 2, “Analysis of postsurgical wound healing.” Please see the correct Fig 2 and Fig 2 caption here.

**Fig 2:**
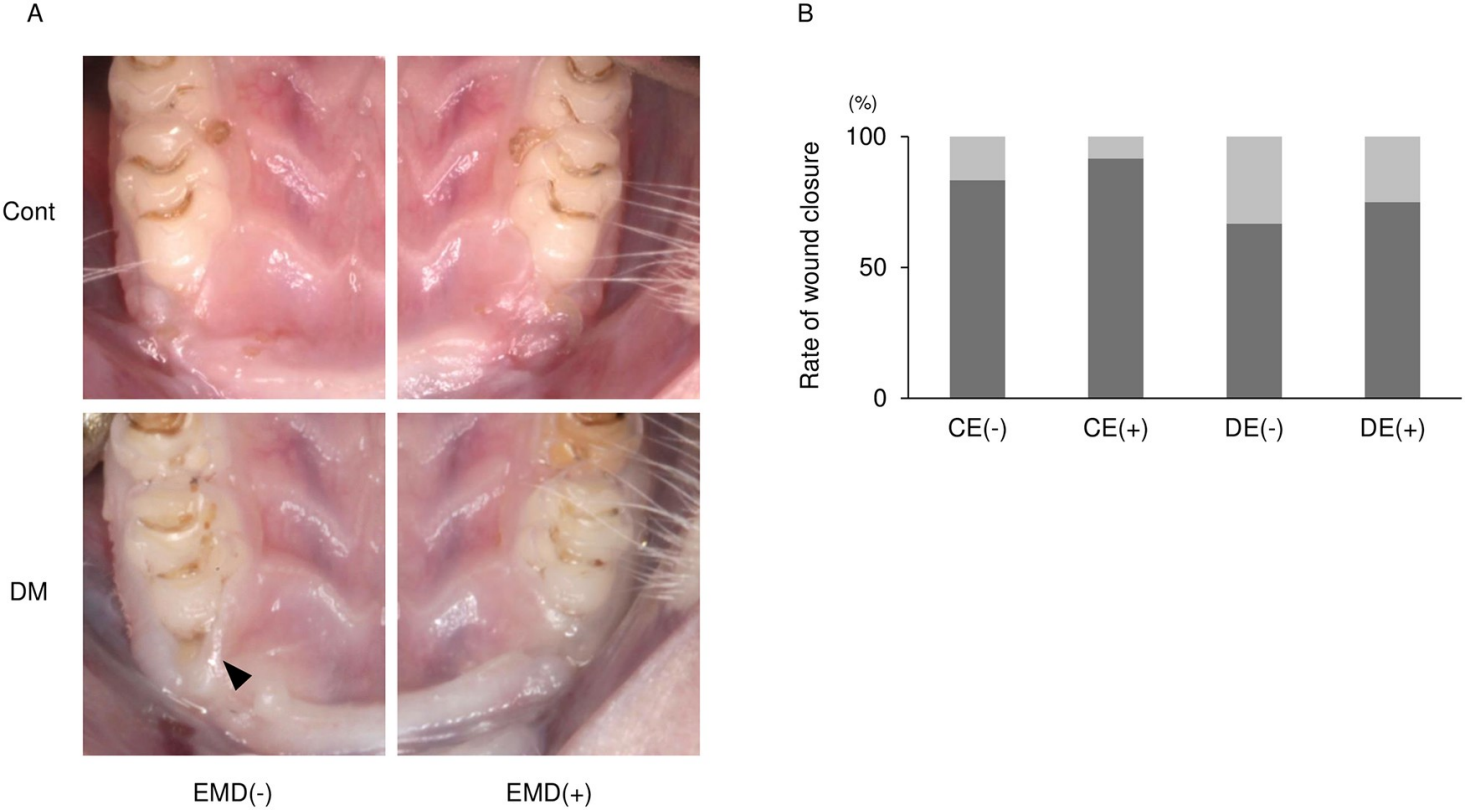
Analysis of postsurgical wound healing. (A) Representative photographs of postsurgical wounds at 7 d after surgery. In the EMD-untreated sites of diabetic rats, wound closure deficiency was observed (arrowheads). (B) Wound closure rates 7 d after surgery among the four groups [control without EMD, CE(-); control with EMD, CE(+); diabetes without EMD, DE(-); and diabetes with EMD, DE(+)]. No statistically significant difference was found between the 4 groups (Fisher’s exact test and binomial test, n = 12).

In the Statistical analysis subsection of the Materials and Methods, there are errors in the second sentence of the first paragraph. The correct sentence is: Fisher’s exact test and binomial test were used to compare the frequency of primary closure in control and diabetic rats.

In the Observation of postsurgical healing subsection of the Results, there are errors in the first and second sentences of the first paragraph. The correct sentences are: At 7 days after surgery, wound closure rates were higher in control rats than in DM rats (not significant). Wound closure rates were higher at the EMD-treated sites of both control and diabetic rats than at untreated sites (not significant).

In the Discussion section, there is an error in the fourth sentence of the fourth paragraph. The correct sentence is: The reduced bone regeneration observed in diabetic animals, although statistical significance was not observed, is attributed to the fact that primary closure is a crucial factor for tissue regeneration.

## References

[1] Takeda K, Mizutani K, Matsuura T, Kido D, Mikami R, Noda M, et al (2018) Periodontal regenerative effect of enamel matrix derivative in diabetes. PLoS ONE 13(11): e0207201 10.1371/journal.pone.0207201 30439990 PMC6237339

