# Supplementary figures and images for: Periodontal regenerative effect of enamel matrix derivative in diabetes
Source: PLoS One. 2018 Nov 15;13(11):e0207201. doi: 10.1371/journal.pone.0207201 (PMC6237339; doi:10.1371/journal.pone.0207201)

## Slide 1
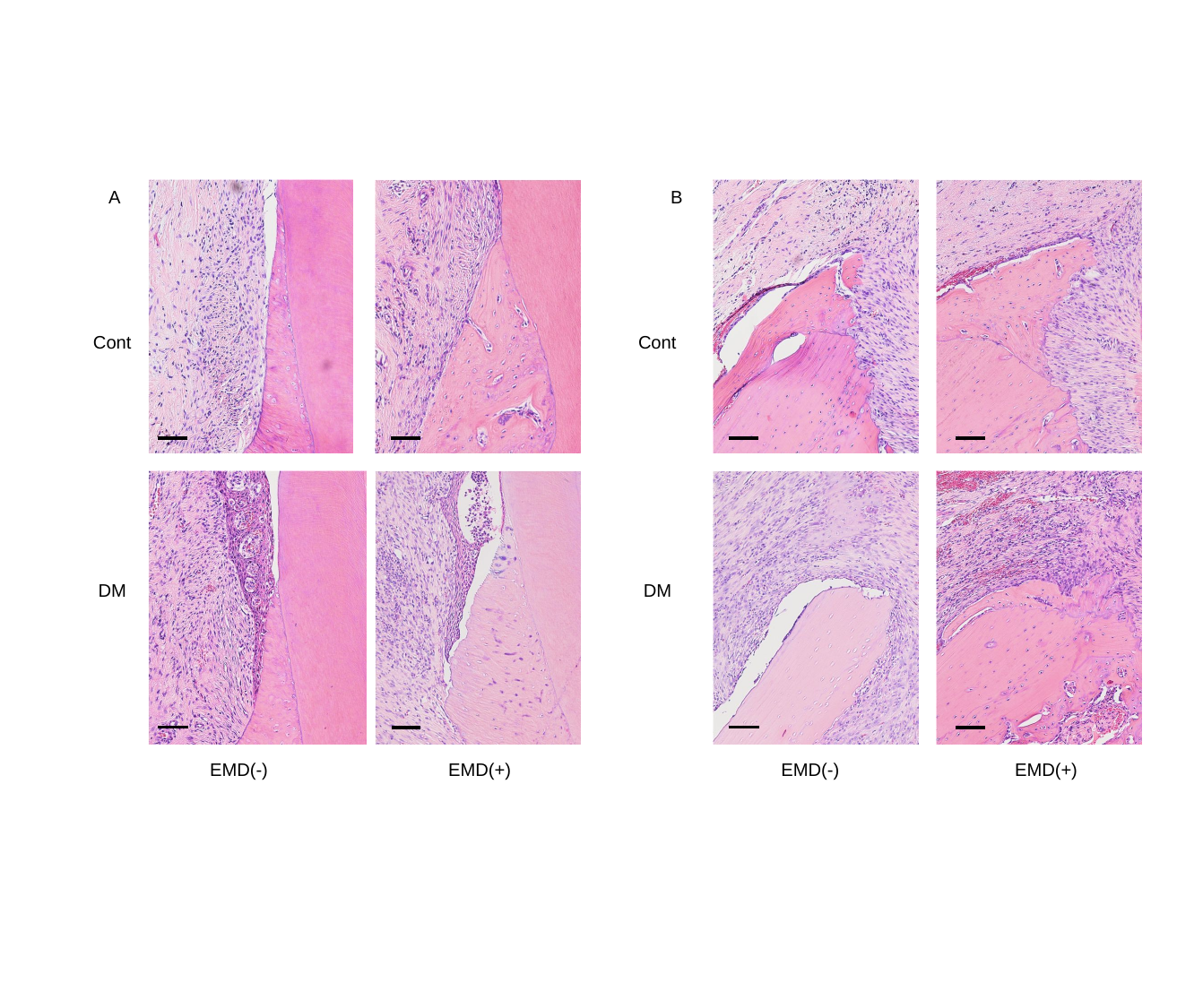

A
B
Cont
Cont
DM
DM
EMD(-)
EMD(+)
EMD(-)
EMD(+)

Supplement: S1 Fig — (A) Representative photographs of histological analysis of the four groups [control without enamel matrix derivative [EMD] and without the cement enamel [CE(-)]; control with EMD, CE(+); diabetes without EMD, DE(-); and diabetes with EMD, DE(+)]. Higher magnification images of the interface shown in Fig 3, focusing on the coronal ends of newly formed cementum. Hematoxylin–eosin staining, magnification 100×. Scale bar: 10 μm. (D) Higher magnification images of the interface shown in Fig 3, focusing on the coronal ends of newly formed bone. Hematoxylin–eosin staining, magnification 100×. Scale bar: 10 μm. (PPTX) [file pone.0207201.s001.pptx]

## Slide 1
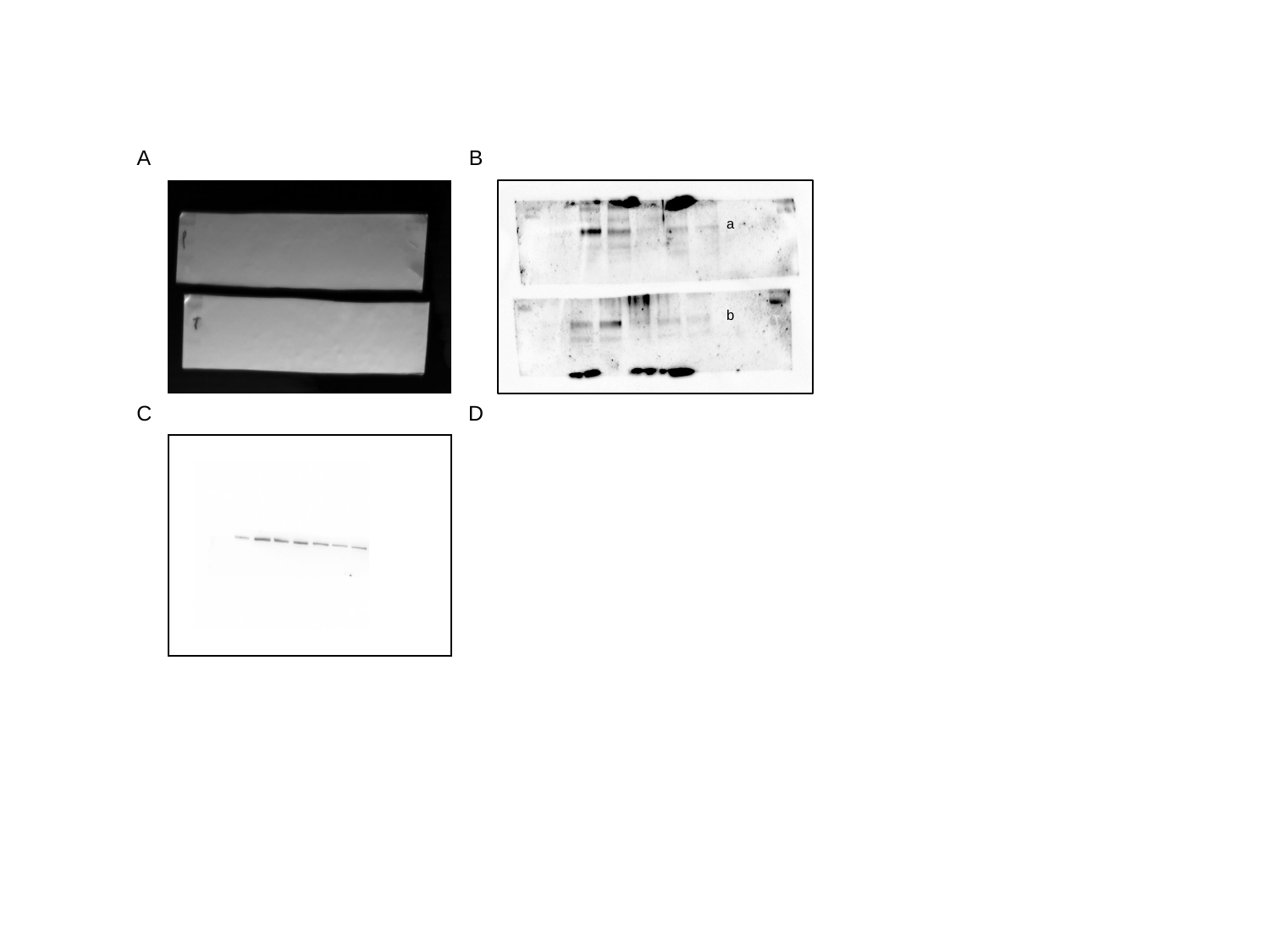

A
B
a
b
D
C

Supplement: S3 Fig — The membrane fragment including the 60 kD band was treated with total and phosphorylated Akt. B-a. Membrane in Fig 7 as p-Akt, B-b Membrane in Fig 7 as t-Akt. C. Membrane in Fig 7 as β-actin. (PPTX) [file pone.0207201.s003.pptx]

## Slide 1
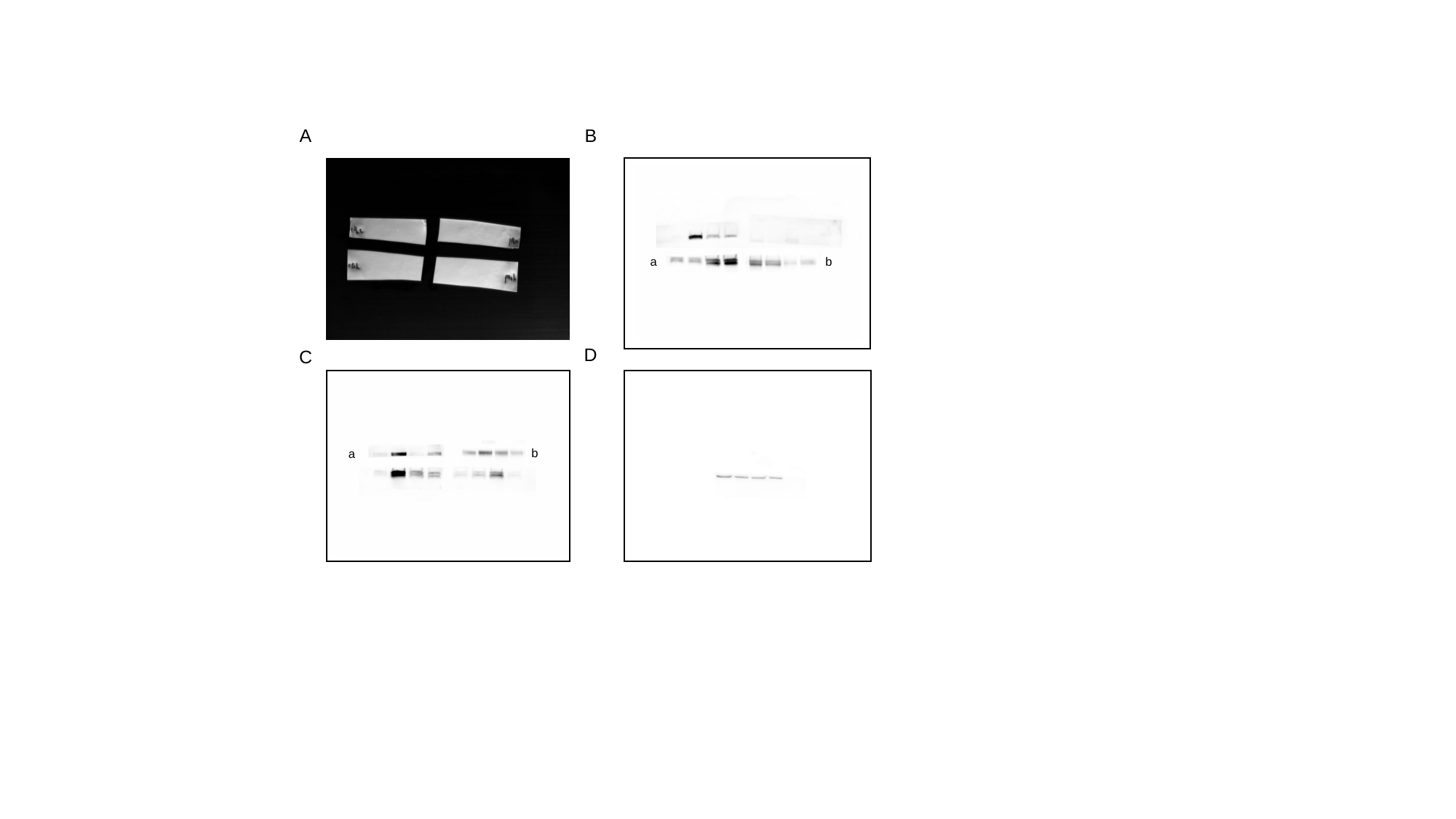

A
B
a
b
D
C
b
a

Supplement: S4 Fig — The membrane was separated at 50 kD. The membrane fragment including the 60 kD band was treated with total and phosphorylated Akt. The other fragment including at 42,44, and 45 kD fragments was treated with total and phosphorylated Erk1/2 and β-actin. A. Original membrane in S2 Fig, B-a. Membrane in S2 Fig as t-Erk1/2, B-b. Membrane in S2 Fig as p-Erk1/2, C-a. Membrane in S2 Fig as t-Akt, C-b Membrane in S2 Fig as p-Akt. D. Membrane in S2 Fig as β-actin. (PPTX) [file pone.0207201.s004.pptx]
