# Supplementary material for: Periodontal regenerative effect of enamel matrix derivative in diabetes
Source: PLoS One. 2018 Nov 15;13(11):e0207201. doi: 10.1371/journal.pone.0207201 (PMC6237339; doi:10.1371/journal.pone.0207201)

**S2_Fig. Analysis of insulin signaling pathway*.***

(A) Effect of insulin on Akt and Erk1/2 phosphorylation in gingival fibroblasts maintained in Cont and HG medium. Representative immunoblots of lysates from insulin-treated and -untreated gingival fibroblasts are shown. (B) Akt and Erk1/2 phosphorylation was quantified by densitometry and expressed as a percentage of phosphorylation in insulin-untreated gingival fibroblasts. Insulin-induced Akt phosphorylation was significantly decreased in HG medium, but Erk1/2 activation was increased. Data are presented as means ± SD. **p* < 0.05 (Tukey-Kramer test). These findings were confirmed in three independent experiments.


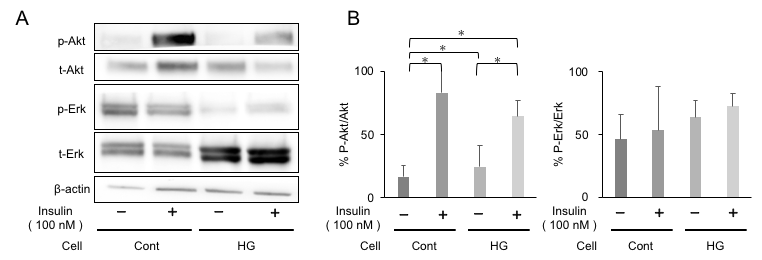

Supplement: S2 Fig — (A) Effect of insulin on Akt and Erk1/2 phosphorylation in gingival fibroblasts cultured in Cont and HG medium. Representative immunoblots of lysates from insulin-treated and -untreated gingival fibroblasts are shown. (B) Akt and Erk1/2 phosphorylation was quantified via densitometric analysis and expressed as a percentage of phosphorylation in insulin-untreated gingival fibroblasts. Insulin-induced Akt phosphorylation was significantly decreased in the HG medium; however, Erk1/2 phosphorylation was increased. Data are presented as mean ± SD values. *p < 0.05 (Tukey-Kramer test). These findings were confirmed in three independent experiments. (DOCX) [file pone.0207201.s002.docx]
