# Supplementary material for: Periodontal regenerative effect of enamel matrix derivative in diabetes
Source: PLoS One. 2018 Nov 15;13(11):e0207201. doi: 10.1371/journal.pone.0207201 (PMC6237339; doi:10.1371/journal.pone.0207201)
Supplement: S1 Table — (DOCX) [file pone.0207201.s005.docx]

**Table 1. Primer sequences are listed.**

| Gene | Forward primer(5'-3') | Reverse primer(5'-3') | GenBank accession no. |
| --- | --- | --- | --- |
| *Vegf* | TGGACCCTGGCTTTACTGCTG | GGCAATAGCTGCGCTGGTAGA | NM_001287114.1 |
| *Collagen type 1* | GCGAAGGCAACAGTCGAT | CTTGGTGGTTTTGTATTCGATGAC | NM_053304.1 |
| *Runx2* | AAGCCTTCCTGATGTGCCAC | GCTCCGGCCTACAAATCTCA | XM_006247374.3 |
| *Tnf-α* | CGTCAGCCGACCATTTC | CCTGTGTGCCCCT | XM_006250947.3 |
| *Il-6* | TTACTCCTTGGAGGCCATGT | AGGACCAGGTTGTCTCCTGT | XM_017593963.1 |
| *Gapdh* | TTACTCCTTGGAGGCCATGT | AGGACCAGGTTGTCTCCTGT | NC_005103.4 |
