# Supplementary material for: Periodontal regenerative effect of enamel matrix derivative in diabetes
Source: PLoS One. 2018 Nov 15;13(11):e0207201. doi: 10.1371/journal.pone.0207201 (PMC6237339; doi:10.1371/journal.pone.0207201)
Supplement: S2 Table — (DOCX) [file pone.0207201.s006.docx]

**Table 2. Body weight, fasting blood glucose level, plasma insulin level, and urine 8-OHdG level in rats changed due to streptozotocin treatment (60 mg/kg)**

|  |  | Cont(n=18) | DM(n=18) | Significance |
| --- | --- | --- | --- | --- |
| Before diabetes induction | Body weight (g) | 113.9±3.0 | 113.4±6.7 | NS |
|  | Fasting blood glucose (mg/dl) | 145.4±9.8 | 153.7±25.8 | NS |
| 2 months after diabetes induction | Body weight (g) | 287.3±39.7 | 185.9±35.6 | p<0.01 |
|  | Fasting blood glucose (mg/dl) | 111.2±12.7 | 421.9±59.7 | p<0.01 |
|  | Insulin(µg/L) | 80.4±12.5 | 1.0±0.1 | p<0.05 |
