# Supplementary material for: Periodontal regenerative effect of enamel matrix derivative in diabetes
Source: PLoS One. 2018 Nov 15;13(11):e0207201. doi: 10.1371/journal.pone.0207201 (PMC6237339; doi:10.1371/journal.pone.0207201)
Supplement: S3 Table — (DOCX) [file pone.0207201.s007.docx]

**Table 3. Statistical results of histological analysis.**

| Parameter | Comparison | *p* value |
| --- | --- | --- |
| CEJ-bone bottom | CE(-), CE(+) vs DE(-), DE(+)  CE(+), DE(+) vs CE(-), DE(-) | F(1,10) = 0.08, *p* = 0.79, NS  F(1,10) = 0.01, *p* = 0.92, NS |
| CEJ-junctional epithelium | CE(-), CE(+) vs DE(-), DE(+)  CE(+), DE(+) vs CE(-), DE(-) | F(1,10) = 48.38, *p* = 0.001  F(1,10) = 0.14, *p* = 0.71, NS |
| New cementum-bottom | CE(-), CE(+) vs DE(-), DE(+)  CE(+), DE(+) vs CE(-), DE(-) | F(1,10) = 15.95, *p* = 0.002  F(1,10) = 5.39, *p* = 0.04 |
| Length of new bone | CE(-) vs CE(+)  CE(+)vs DE(+) | F(1,10) = 30.51, *p* = 0.001  F(1,10) = 10.83, *p* = 0.001 |
| Area of new cementum | CE(-), CE(+) vs DE(-), DE(+)  CE(+), DE(+) vs CE(-), DE(-) | F(1,10) = 7.50, *p* = 0.02  F(1,10) = 18.06, *p* = 0.002 |
| Area of new bone | CE(-), CE(+) vs DE(-), DE(+)  CE(+), DE(+) vs CE(-), DE(-) | F(1,10) = 12.24, *p* = 0.006  F(1,10) = 18.11, *p* = 0.001 |
