# Supplementary material for: Periodontal regenerative effect of enamel matrix derivative in diabetes
Source: PLoS One. 2018 Nov 15;13(11):e0207201. doi: 10.1371/journal.pone.0207201 (PMC6237339; doi:10.1371/journal.pone.0207201)
Supplement: S4 Table — (DOCX) [file pone.0207201.s008.docx]

**Table 4. Statistical results of micro-CT analysis.**

| Parameter | Comparison | *p* value |
| --- | --- | --- |
| BV | CE(-), CE(+) vs DE(-), DE(+)  CE(+), DE(+) vs CE(-), DE(-) | F(1,10) = 8.95, *p* = 0.006  F(1,10) = 11.29, *p* = 0.01 |
| CBV | CE(-), CE(+) vs DE(-), DE(+)  CE(+), DE(+) vs CE(-), DE(-) | F(1,10) = 7.35, *p* = 0.01  F(1,10) = 9.02, *p* = 0.02 |
| BMD | CE(-), CE(+) vs DE(-), DE(+)  CE(+), DE(+) vs CE(-), DE(-) | F(1,10) = 9.81, *p* = 0.006  F(1,10) = 11.77, *p* = 0.009 |
| CBM | CE(-), CE(+) vs DE(-), DE(+)  CE(+), DE(+) vs CE(-), DE(-) | F(1,10) = 7.48, *p* = 0.01  F(1,10) = 9.60, *p* = 0.02 |
